# Acceleration rate of mitral inflow E wave: a novel transmitral doppler index for assessing diastolic function

**DOI:** 10.1186/s12947-016-0067-2

**Published:** 2016-06-10

**Authors:** Roya Sattarzadeh Badkoubeh, Anahita Tavoosi, Mostafa Jabbari, Amir Farhang Zand Parsa, Babak Geraeli, Mohammad Saadat, Farnoosh Larti, Ali Pasha Meysamie, Mehrdad Salehi

**Affiliations:** 1 Cardiology Department of Imam Khomeini Hospital, Tehran University of Medical Sciences, Tehran, Iran; 2 Department of community medicine, Tehran University of Medical Sciences, Tehran, Iran

## Abstract

**Background:**

We performed comprehensive transmitral and pulmonary venous Doppler echocardiographic studies to devise a novel index of diastolic function. This is the first study to assess the utility of the acceleration rate (AR) of the E wave of mitral inflow as a primary diagnostic modality for assessing diastolic function.

**Methods:**

Study group consisted of 84 patients (53 + 11 years) with left ventricle (LV) diastolic dysfunction and 34 healthy people (35 ± 9 years) as control group, who were referred for clinically indicated two-dimensional transthoracic echocardiogram (TTE) during 2012 and 2013 to Imam Hospital. Normal controls were defined as patients without clinical evidence of cardiac disease and had normal TTE. LV diastolic function was determined according to standardized protocol of American Society of Echocardiography (ASE). As our new parameter, AR of E wave of mitral inflow was also measured in all patients. It was represented by the slope of the line between onset of E wave and peak of it. Correlation between AR of E wave and LV diastolic function grade was measured using the Spearman correlation coefficient. Receiver operating characteristic (ROC) curve was used to determine the sensitivity and specificity of AR of E wave in diagnosing LV diastolic dysfunction in randomly selected two-thirds of population then its derived cutoff was evaluated in rest of the population. The institutional review board of the hospital approved the study protocol. All participants gave written informed consent. This investigation was in accordance with the Declaration of Helsinki.

**Results:**

The mean value of AR was 1010 ± 420 cm/s^2^ in patients whereas the mean value for the normal controls was 701 ± 210 cm/s^2^. There was a strong and graded relation between AR of E wave of mitral inflow and LV diastolic function grade (Spearman *P* ≤0.0001, r_s_ =0.69). ROC curve analysis revealed that AR of E wave of mitral inflow =750 cm/s^2^ predicted moderate or severe LV diastolic dysfunction with 89 % sensitivity and 89 % specificity (area under curve [AUC] = 0.903, *P* <0.0001). Application of this cutoff on test group showed 96 % sensitivity and 77 % specificity with AUC = 0.932 and *P* <0.0001.

**Conclusion:**

AR of E wave of mitral inflow could be used for assessment of diastolic function, especially moderate or severe diastolic dysfunction. However, before its clinical application, external validation should be considered.

## Background

Over the past two decades, the prevalence of heart failure due to diastolic dysfunction has been gradually rising. Despite the growing incidence of this disorder, no effective therapies exist to treat the disease, halt its progression or reduce the associated mortality [1]. The assessment of left ventricular (LV) diastolic function and filling pressures is of paramount clinical importance to distinguish this syndrome from other diseases such as pulmonary disease resulting in dyspnea, to assess prognosis, and to identify underlying cardiac disease and its best treatment. LV filling pressures as measured invasively include mean pulmonary wedge pressure or mean left atrial (LA) pressure, and LV end-diastolic pressure [2, 3]. Echocardiography has played a central role in the evaluation of LV diastolic function over the past two decades. Transmitral Doppler echocardiography has been routinely used to identify left ventricular diastolic dysfunction in patients [4, 5]. However, problems related to the complexity of interpreting the transmitral flow profile still exist, and some of the better established clinical indices may need to be re-evaluated for their relevance.

The estimation of LV filling pressures in patients with normal ejection fractions (EF)s is more challenging than in patients with depressed EFs. In this patient group, the ratio of mitral peak velocity of early filling (E) to early diastolic mitral annular velocity (e’), the E/e’ ratio, should be calculated. An average ratio ≤8 identifies patients with normal LV filling pressures, whereas a ratio ≥13 indicates an increase in LV filling pressures. When the ratio is between 9 and 13, other measurements are essential. A pulmonary venous (PV) atrial reversal wave (Ar) duration longer than 30 ms of that of the transmitral A wave, a change in E/A ratio with the Valsalva maneuver of ≥0.5, ratio between isovolumetric relaxation time (IVRT) and the time delay (T_E- e’_) between onset of mitral E and annular e,less than 2 (IVRT/T_E-e’_ <2), pulmonary artery systolic pressure ≥35 mm Hg (in the absence of pulmonary disease), and maximal LA volume ≥34 mL/m^2^ are all indicative of increased LV filling pressures. The presence of ≥2 abnormal measurements increases the confidence in the conclusions [6]. By the way according to literature each of the mentioned criteria has some limitations which cause some difficulties in explanation of echocardiography results.

Therefore, we performed comprehensive transmitral and pulmonary venous Doppler echocardiographic studies to devise a novel index of diastolic function. This is the first study to assess the utility of the acceleration rate and time of the E wave of mitral inflow as a primary diagnostic modality for assessing diastolic function.

## Methods

Study group consisted of 84 patients with LV diastolic dysfunction and 34 healthy people as control group, who were referred for clinically indicated two-dimensional transthoracic echocardiogram (TTE) between 2012 January and 2013 May. The inclusion criterion was presence of LV diastolic dysfunction in TTE. Patients with unstable hemodynamic state, arrhythmia, valvular heart disease, congenital heart disease, constrictive pericarditis or permanent pacemaker implantation were excluded. Normal controls were defined as patients without clinical evidence of cardiac disease and had normal TTE. Clinical data were obtained through a comprehensive review of patient’s medical records.

The institutional review board of Imam Khomeini Hospital which is a tertiary hospital approved the study protocol. All participants gave written informed consent. This investigation was in accordance with the Declaration of Helsinki.

### Standard transthoracic echocardiography

Complete M-mode, two-dimensional and Doppler echocardiogram was performed by two experienced cardiologists according to standardized protocol of American Society of Echocardiography [7, 8] using a commercially available instrument (VIVID 7, GE-Ving Med, Horten, Norway) equipped with a 3.5 MHz transducer. Making use of the modified Simpson method, LV ejection fraction was measured at the apical four-chamber view.

### Assessment of diastolic function

Mitral inflow was assessed from the apical 4-chamber view with pulsed wave Doppler by placing a 1–2 mm sample volume between the tips of the mitral leaflets during diastole. From the mitral inflow profile, the E- and A-wave velocity, E-deceleration time (DT), A-wave duration, and *E*/*A* velocity ratio were measured. Pulmonary venous velocities were obtained from the same window with the sample volume placed 1 cm into the right upper pulmonary vein. The flow velocities were recorded, the ratio of systolic to diastolic flow (*S*/*D* ratio) was calculated and duration of atrial reversal flow was measured. Doppler tissue imaging was used to measure *E' * and *A'* velocities by placing a 1–2 mm sample volume in the septal and lateral mitral annulus.

LV diastolic function was determined using standard echocardiographic parameters including *E*/*A* velocity ratio, E-DT, PV atrial reversal velocity and duration, PV *S*/*D* ratio, and mitral *E*/*E' * ratio. Diastolic function is graded as normal, abnormal relaxation (Grade I), pseudonormal (Grade II), and restrictive (Grade III) as described previously. Pseudonormal (Grade II) is differentiated from normal by having (i) PV atrial reversal duration longer than mitral A duration by 30 ms; or (ii) peak PV atrial reversal velocity >35 cm/s; or (iii) mitral *E*/*E'* >12 (lateral annulus) or >15 (septal annulus) [2, 6].

### Acceleration rate of E wave of mitral inflow (AR)

**Fig. 1:**
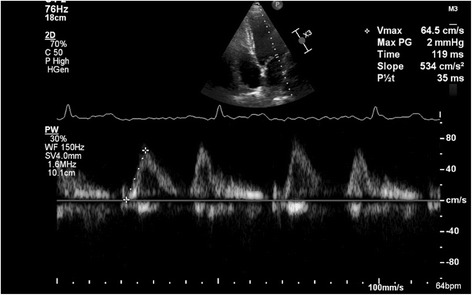
Acceleration rate of E wave of mitral inflow

As our new parameter acceleration rate and time of E was also measured in all patients. Acceleration rate of E (cm/sec2) was represented by the slope of the line between an anchored point and a crosshair (Fig. 1). This linear measurement was made on the velocity spectrum. Acceleration time (AT) of E was measured from onset to peak of E These recordings were shown on a strip chart with a sweep speed of 100 mm/s to determine correct temporal observations. Measurements were performed off line by an independent observer who had no knowledge of the Doppler or Tissue Doppler findings. At least three measurements were taken of each parameter and these were averaged.

### Statistical analysis

SPSS release 21.0 statistical package was used for data analysis. All values were expressed as mean ± SD. Correlation between AR and AT of E wave and LV diastolic function grade was measured using the Spearman correlation coefficient. Multivariate Logistic Regression analysis was also done to adjust the age and gender effect. Receiver operating characteristic (ROC) curve was used to determine the sensitivity and specificity of AR of E wave in diagnosing LV diastolic dysfunction and elevated left ventricle diastolic pressure (LVDP). Study population randomly assigned into two groups with 2:1 ratio. ROC curve was performed in randomly selected two-thirds of population (derivation group), then the derived cutoff was evaluated in the rest of the population (Test group). Therefore, ROC curve analysis was performed two times. First, we recoded the “diastolic function” into a dichotic variable just based on presence or absence of any degree of diastolic dysfunction (mild, moderate, and severe). Second, we recoded the “diastolic function” into another dichotic variable, this time based on presence or absence of elevated LVDP (moderate and severe diastolic dysfunction).

## Result

The study population consisted of 84 adult patients (64 % men), mean age 53 ± 11(range, 20–70 years) and 34 normal controls, who were referred for clinically indicated two-dimensional echocardiogram. They were referred because of the following reasons: dyspnea/peripheral edema/congestive heart failure (48 %), cerebrovascular accident (12 %), preoperative assessment (10 %), coronary artery disease (9 %), and others (21 %).

The baseline clinical and echocardiographic characteristics are listed in Table 1.

**Table 1:** Echocardiographic parameters of the study population

	Normal (*n* = 34)	Grade I (*n* = 24)	Grade II (*n* = 39)	Grade III (*n* = 21)	Total (*n* = 118)
Age (year)	34.6 ± 9.1	53.2 ± 7.2	54.7 ± 11.7	48.9 ± 12.9	47.5 ± 13.4
Men (%)	82	46	69	76	69
Left ventricle ejection fraction (Simpson) (%)	55.7 ± 4	52.4 ± 4.3	37.9 ± 2.9	27.6 ± 12.4	44.3 ± 13.8
Mitral peak E velocity (m/s)	0.7 ± 0.1	0.50 ± 0.1	0.7 ± 0.2	1.04 ± 0.2	0.7 ± 0.2
Mitral peak A velocity (m/s)	0.5 ± 0.09	0.7 ± 0.1	0.6 ± 0.1	0.3 ± 0.1	0.6 ± 0.2
Mitral E/A ratio	1.4 ± 0.3	0.7 ± 0.1	1.2 ± 0.4	2.9 ± 0.7	1.5 ± 0.8
Mitral deceleration time (ms)	182 ± 30	236 ± 23	170 ± 51	108 ± 38	176 ± 55
Pulmonary venous systolic forward flow velocity (m/s)	0.66 ± 0.08	0.72 ± 0.12	0.54 ± 0.17	0.35 ± 0.14	0.58 ± 0.18
Pulmonary venous diastolic forward flow velocity (m/s)	0.54 ± 0.08	0.45 ± 0.07	0.64 ± 0.18	0.75 ± 0.23	0.59 ± 0.18
Pulmonary venous atrial reversal velocity (m/s)	0.23 ± 0.03	0.27 ± 0.05	0.36 ± 0.08	0.37 ± 0.5	0.30 ± 0.08
Tissue Doppler imaging septal E' (cm/s)	10.5 ± 2.2	6 ± 0.9	6 ± 2.2	5.1 ± 1.7	7.1 ± 2.9
Tissue Doppler imaging lateral E' (cm/s)	13.4 ± 3.0	8.2 ± 1.7	7.3 ± 3.0	6.8 ± 2.2	9.1 ± 3.8
Tissue Doppler imaging septal A' (cm/s)	7.6 ± 1.6	9.5 ± 1.6	6.7 ± 2.4	3.8 ± 1.7	7 ± 2.6
Tissue Doppler imaging lateral A' (cm/s)	7.3 ± 2.2	10.3 ± 2.3	5.7 ± 2.3	3.5 ± 1.1	6.7 ± 3.1
E/E'	7.2 ± 1.7	9.7 ± 2.5	13.9 ± 5.6	22.3 ± 8.2	12.6 ± 7.2
Color M-mode flow propagation velocity (cm/s)	61.9 ± 7.9	49.8 ± 3.4	41.6 ± 6.6	36.5 ± 6.1	48.4 ± 11.5
Left atrium volume (cm^3^)	42 ± 11.7	46.2 ± 9.2	88.4 ± 28.3	107 ± 29.7	69.8 ± 24.2

**Fig. 2:**
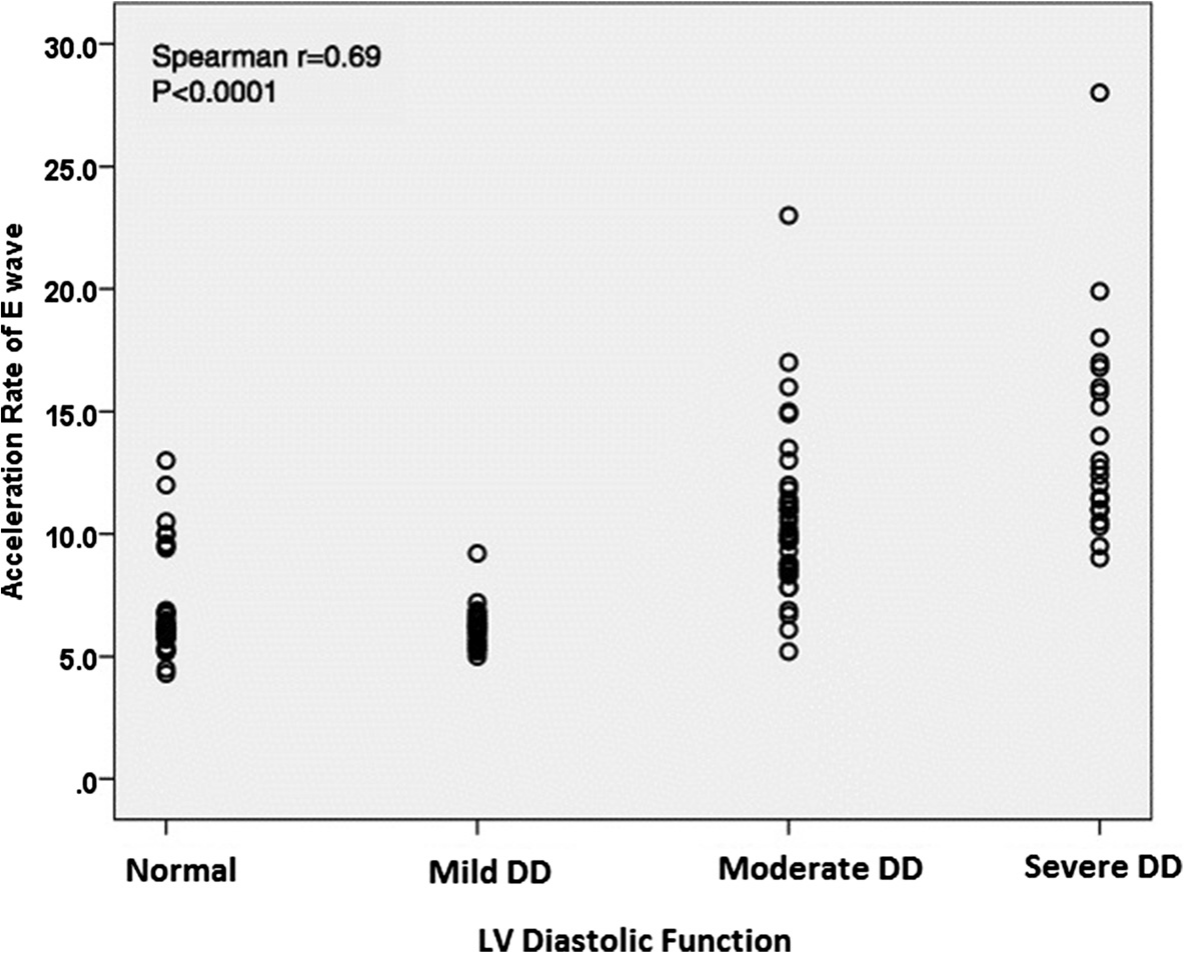
Relationship between acceleration rate of E wave of mitral inflow and LV diastolic function grade

**Fig. 3:**
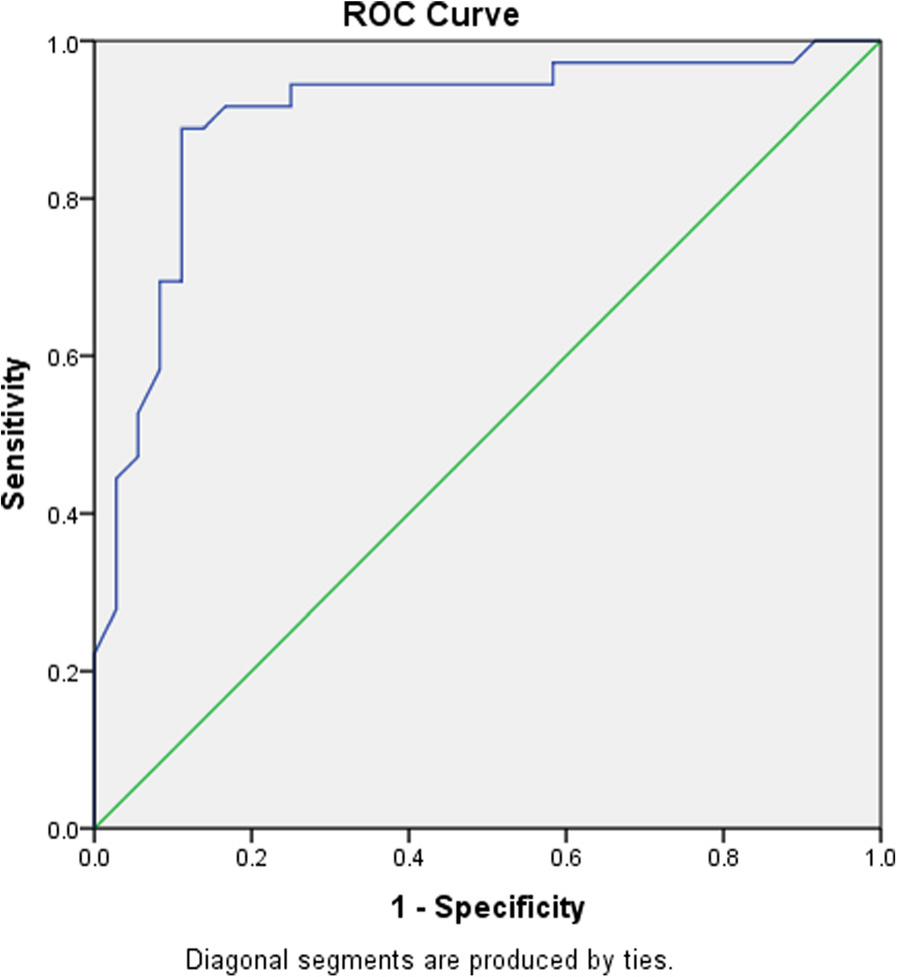
Receiver operating characteristic curve for the detection of Moderate or Severe diastolic dysfunction using acceleration rate of E wave of mitral inflow (area under curve 0.903, *P* <0.0001)

The mean value of E acceleration time and E acceleration rate were 85.6 ± 19.1 ms, and 1010 ± 420 cm/s^2^ in case group respectively, whereas these mean values for the normal controls were 96.7 ± 15.1 ms, and 701 ± 210 cm/s^2^respectively. The mean values of AT and AR according to grade of LV diastolic dysfunction is showed in Table 2. There was a strong and graded relation between AR of E wave of mitral inflow and LV diastolic function grade (Spearman *P* ≤0.0001, r_s_ =0.69) (Fig. 2). The Logistic Regression Analysis analysis showed that AR of E could predict the diastolic dysfunction after adjustment for age and gender (*P* = 0.001). Receiver operating characteristic (ROC) curve analysis in “derivation group” revealed that AR of E wave of mitral inflow =655 cm/s^2^ predicted presence of LV diastolic dysfunction with 71 % sensitivity and 65 % specificity (area under curve [AUC] = 0. 0.727, *P* = 0.003). Application of this cutoff on test group showed 84 % sensitivity and 64 % specificity with AUC = 0.800 and *P* = 0.001. When considering only Grade II and III (moderate and severe) diastolic dysfunction, an AR of 750 cm/s^2^ predicted at least moderate diastolic dysfunction with 89 % sensitivity and 89 % specificity (area under curve 0.903, *P* <0.0001) (Fig. 3, Table 3). Application of this cutoff on test group showed 96 % sensitivity and 77 % specificity with AUC = 0.932 and *P* <0.0001. AR of E = 1250 cm/s^2^ was 100 % specific for the detection of elevated LVDP (moderate or severe diastolic dysfunction) but with a low sensitivity of 22 %.

**Table 2:** Acceleration rate and time of E wave by diastolic function grade

	Normal (*n* = 34)	Grade I (*n* = 24)	Grade II (*n* = 39)	Grade III (*n* = 21)	Total (*n* = 118)
Acceleration time (ms)	96.7 ± 15.1	95.7 ± 16.7	83 ± 18.9	79.1 ± 18.5	88.8 ± 18.7
Acceleration rate (cm/s^2^)	701 ± 210	620 ± 90	1060 ± 330	1400 ± 440	930 ± 400

**Table 3:** Positive Predictive Value (PPV), Negative Predictive Value (NPV), sensitivity and specificity of acceleration rate of E to detect moderate or severe diastolic dysfunction

		Moderate or severe diastolic dysfunction		
		*No*	*Yes*	
Acceleration rate of E	*<750*	*32*	*4*	*NPV = 89 %*
	*≥750*	*4*	*32*	*PPV = 89 %*
		*Spe = 89 %*	*Sen = 89 %*	

## Discussion

The present study showed that AR of E wave of mitral inflow has a strong and graded relation to the LV diastolic function grade, and could be used especially to identify moderate or severe LV diastolic dysfunction. This result was predictable because according to Newton’s second law there is a direct relationship between acceleration and pressure. E wave acceleration is directly determined by LA pressure and inversely related to myocardial relaxation [9]. Thomas and weyman [10] demonstrated in a mathematical model of LV filling that AR is greatly affected by changes in left atrial pressure compared with peak velocity, peak deceleration, and the total integral of the inflow velocity.

Despite the theoretical observations by Thomas and Weyman [10], AR has not been used clinically for estimation of LA pressure. There are two studies which reported AT and AR as parameters which can be useful for assessing left ventricular diastolic function in patients with diabetes or coronary artery diseases [11, 12]. There is also an experimental study on mice which reported AT of E wave was sensitive for detecting early stages of diastolic function, and appeared to add incremental value over that provided by the E/A ratio and IVRT for detecting later stages of diastolic dysfunction in murine models [13]. To our knowledge none of these studies reported a cut of value for AR of E wave to predict LV diastolic dysfunction. However in 1996 Nagueh et al. showed that peak AR ≥1900 cm/s^2^ had a 77 % sensitivity and 94 % specificity for left ventricle diastolic pressure (LVDP) >15 mmHg in patients with atrial fibrillation(AF) [14]. In our study we showed that by simple measurement of AR of E of mitral valve we might be able to identify patients with elevated LVDP. This application could be clinically important and valuable because it might explain patients’ symptoms in some conditions. The cut off value of 750 cm/s^2^ had a suitable sensitivity and specificity for elevated LVDP according to diastolic function grade II or more. And the cut off value of 1250 cm/s^2^ had 100 % specificity for diastolic dysfunction of grade II or more. The difference in our cut off value and the previous one could have three reasons. First, in our study we used AR which conventionally could be measured by every machine of echocardiography, but Naughueh et.al used a computer software to measure peak of AR. Second the method of estimation of elevated LVDP was different in two studies. In our study we postulated that presence of diastolic dysfunction of grade II or more would be associated with elevated LVDP, however Naughueh et al. directly measured LVDP or LA pressure in their patients. Third, all of our patients had sinus rhythm, but the rhythm of patients in that study was AF.

### Limitation

The limitations of our study include its relatively small size. The ‘normal controls’ are included on the basis of absence of a history of cardiovascular disease and a normal resting two-dimensional echocardiogram. Stress tests were not performed to rule out occult coronary artery disease. We used previously published Doppler echocardiographic referenced standards to define the different grades of LV diastolic function. Cardiac catheterization was not performed to evaluate LV diastolic function. However, these reference standards were previously validated with cardiac catheterization and are widely accepted as standards for classification of LV diastolic function grade. Although the results of our study about AR are promising, before its clinical application external validation should be considered. Further studies with invasive haemodynamic measurements are needed to show if AR measurement increase the diagnostic accuracy of LV diastolic dysfunction and elevated LA pressure with respect to validated echo-Doppler parameters.

## Conclusion

AR of EARE wave of mitral inflow could be used for assessment of diastolic function, especially moderate to severe diastolic dysfunction. The cutoff value of 750 cm/s2 could deserve as suitable cutoff point with 89 % sensitivity and 89 % specificity in detection of moderate to severe diastolic dysfunction. Before its clinical application external validation should be considered.
