# Erratum to: ‘Acceleration rate of mitral inflow E wave: a novel transmitral doppler index for assessing diastolic function’

**DOI:** 10.1186/s12947-016-0070-7

**Published:** 2016-07-26

**Authors:** Roya Sattarzadeh, Anahita Tavoosi, Mostafa Jabbari, Amir Farhang Zand Parsa, Babak Geraiely, Mohammad Saadat, Farnoosh Larti, Ali Pasha Meysamie, Mehrdad Salehi

**Affiliations:** 1 Cardiology Department of Imam Khomeini Hospital, Tehran University of Medical Sciences, Tehran, Iran; 2 Department of Community Medicine, Tehran University of Medical Sciences, Tehran, Iran

## Erratum

Unfortunately, the original version of this article [1] contained an errors. Two corrections are with the author’s name. In the metadata of the article Roya Sattarzadeh Badkoubeh was published instead of Roya Sattarzadeh. The second author’s name recorded incorrectly was Babak Geraeli. This should be corrected to Babak Geraiely.
